# Cardiac Autonomic Function Following Bilateral and Unilateral Upper Body Acute Resistance Exercise

**DOI:** 10.3390/ijerph19106077

**Published:** 2022-05-17

**Authors:** Erica M. Marshall, Jason C. Parks, Emily K. Erb, Stacie M. Humm, J. Derek Kingsley

**Affiliations:** 1 Exercise Science, Florida Southern College, Lakeland, FL 33801, USA; 2 Kinesiology, State University of New York at Cortland, Cortland, NY 13045, USA; jason.parks@cortland.edu; 3 Exercise Science and Exercise Physiology, Kent State University, Kent, OH 44242, USA; eerb4@kent.edu (E.K.E.); shumm2@kent.edu (S.M.H.); jkingsle@kent.edu (J.D.K.)

**Keywords:** heart rate variability, baroreflex sensitivity, free weights

## Abstract

The purpose of this study was to compare cardiac autonomic responses following bilateral and unilateral upper-body (UB) acute resistance exercise (ARE). In total, 14 individuals were assessed for markers of cardiac autonomic responses via heart rate variability (HRV) and baroreflex sensitivity (BRS) at rest and at 10- and 30-min following ARE. Logarithmically transformed (ln) HRV measures included: total power (ln TP), high-frequency power (ln HF power), low-frequency power (ln LF power), sympathovagal balance (ln LF: HF), and the square root of the mean squared differences of successive R-R intervals (ln RMSSD). BRS was assessed using the sequence method. Two-way repeated measures ANOVAs were used to analyze effects of UB ARE (bilateral, unilateral) across time (Rest, 10, and 30 min). There were no significant (*p* > 0.05) interactions. However, there were significant (*p* ≤ 0.05) main effects of time such that ln TP, ln HF power, ln RMSSD, and BRS decreased and did not recover within 30 min compared to Rest for both conditions. Collectively, this study suggests that bilateral and unilateral UB ARE yielded similar reductions, for at least 30 min, in respect to vagal measures of HRV and BRS.

## 1. Introduction

Acute resistance exercise (ARE) reportedly alters cardiac autonomic nervous system (ANS) function during the recovery period [1,2,3]. Specifically, ARE attenuates vagal tone (i.e., parasympathetic nervous system) [2,3,4]. Despite the fact that these reductions have been associated with all-cause mortality [5] and sudden cardiac death in normotensive populations [6]. Modalities or interventions that could attenuate these changes are lacking in the literature. Interestingly, in a study by Moreira, et al. [7], upper body (UB) ARE performed unilaterally had an attenuated heart rate (HR) and systolic BP (SBP) response compared to bilateral UB ARE in young healthy individuals. Prospectively, unilateral UB ARE may improve vagal recovery in a normotensive population, but this is unknown. While it has been demonstrated that cardiac ANS responses are muscle mass dependent [8,9], it is unclear if increased core muscle involvement with unilateral movements, which aids in stabilization, would lead to similar responses compared to bilateral UB ARE. Thus, more research with this modality is warranted to ascertain the effects of bilateral versus unilateral resistance exercise on vagal recovery in a normotensive population.

Cardiac ANS function of the heart is quantified using heart rate variability (HRV), which involves linear analysis of the heart rate (HR) and corresponding R-R intervals on the electrocardiogram (ECG) in frequency and time domains [10]. Frequency domain measures include: total power (TP), the variance of R-R intervals and an indication of cardiac ANS function; high-frequency (HF) power, a measure of vagal activity; low-frequency (LF) power, a measure of both vagal and sympathetic activity [10]; and the LF to HF ratio (LF:HF ratio), a measure of sympathovagal dominance [11]. The time domain measure includes the square root of the mean squared differences of successive R-R intervals (RMSSD), indicative of vagal activity [10]. Further, baroreflex sensitivity (BRS), which regulates changes in the R-R interval in relation to blood pressure (BP), can also be used as a measure of cardiac ANS function. The baroreflex itself operates on a negative feedback loop to ANS control centers in the medulla, and serves to regulate BP [12]. Reductions in BRS indicate a disruption in the negative feedback loop, which may elicit unfavorable alterations in left ventricular mechanics and further increased risk of arrythmia [13,14].

### Purpose of Study

Current literature suggests that cardiac ANS function, specifically vagal activity is depressed following UB ARE as indicated by reductions in TP [2,3], HF power [2,3,4,15], increases in LF:HF ratio [2], and decreases in the RMSSD [2,3,4,15] for at least 30 min. The effects of UB ARE on BRS have yet to be fully elucidated. Therefore, the purpose of the present study was to compare the effects of unilateral and bilateral UB ARE on cardiac ANS function as measured by HRV and BRS at rest, as well as 10- and 30-min during recovery. The primary hypotheses for the present study were that following unilateral UB ARE, measures of HRV, namely TP, HF power, the LF:HF ratio, and the RMSSD would be augmented at similar time points and also recovered by 30 min compared to bilateral UB ARE. Similarly, it was hypothesized that BRS would be augmented at similar time points and also recovered by 30 min following unilateral UB ARE compared to bilateral UB ARE.

## 2. Materials and Methods

### 2.1. Participants

In total, 14 (*n* = 14; Men = 7, Women = 7) healthy, moderately active individuals participated in this study. Physical activity status was determined using the Lipid Research Clinic questionnaire [16], which consisted of 4 questions and used a 4-point scoring method to determine physical activity status [16]. Participants were asked to rate their physical activity at work and at home compared to age and sex-matched peers, participation in strenuous exercise, and whether they performed physical activity at least 3 times per week. Moderately active individuals were defined as those who performed strenuous exercise at least 3 days per week and were somewhat more/less physically active at work and home, or about the same compared to age and sex-matched peers. Participants were excluded if they had a recent smoking history (<6 months), obesity (defined as a BMI ≥ 30 kg/m^2^), hypertension (resting systolic blood pressure (SBP) ≥ 130 mmHg or diastolic blood pressure (DBP) ≥ 80 mmHg), orthopedic problems, open wounds, cancer, known metabolic disease, cardiovascular disease, or renal disease, in addition to if they were using medications or supplements known to affect HR or BP. All forementioned criteria were assessed during the orientation via the Physical Activity Readiness Questionnaire (PARQ) and Health History Questionnaire. All participants signed a written informed consent. This study was approved by the Institutional Review Board (IRB#: 19-185) and corresponded to the Declaration of Helsinki.

### 2.2. Study Design

**Figure 1:**
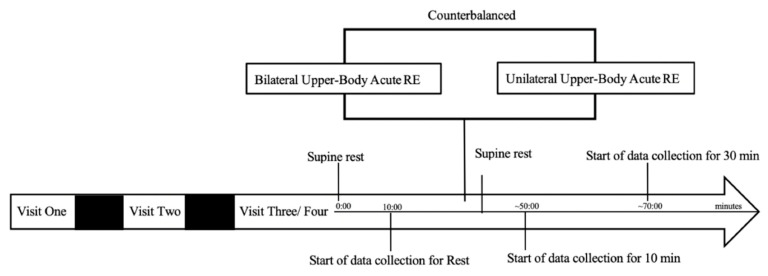
Timeline of data collection procedures.

Following consent and orientation, participants completed 4 additional visits. During Visit 1, participants were first assessed for anthropometrics and body composition followed by maximal strength testing on the bilateral dumbbell (DB) bench press and bilateral DB biceps curl for a 5-repetition maximum (RM) and 10 RM, respectively. Visit 2 consisted of maximal strength testing on the unilateral DB bench press and unilateral DB biceps curl. Finally, Visits 3 and 4 consisted of cardiac ANS function data collection at rest and at 10- and 30-min during recovery. Bilateral and unilateral UB ARE were completed in a counterbalanced format. Both data collection visits were performed at the same time of day (±1 h) and occurred between the hours of 6 a.m. to 12 p.m. in order to account for diurnal variation. All visits were at least 72 h apart (see Figure 1). Participants were asked to abstain from caffeine, alcohol, and strenuous exercise for at least 24 h, and food for at least 3 h prior to testing. All women were tested during the early follicular phase of the menstrual cycle (Day 1–7) denoted by the start of the menses.

### 2.3. Visits 1 and 2

#### Anthropometric and Strength Assessment

Participants’ height and weight were assessed using a stadiometer and balance beam scale (Detecto 448; Cardinal Scale Manufacturing, Web City, MO, USA), respectively. Height was measured to the 0.1 cm (cm) and weight to the nearest 0.1 pound (lb) and then converted to kilograms (kg) to compute body mass index (BMI). Body composition was measured by 7 site skinfold measurement (Lange; Beta Technology, Santa Cruz, CA, USA). Generalized skinfold equations were used to determine body density for men [17] and women [18]. The Brozek equation was used to calculate percent body fat [19].

Participants first completed a 5-min warm-up on the cycle ergometer (Schwinn Air Dyne; Boulder, Colorado). Following, maximal strength testing at the 5 RM on the DB bench press and 10 RM on the DB biceps curl was carried out utilizing previously described methods [20]. Specifically, participants were given a warm-up at a weight they could easily move for ~10 repetitions at 50% of their estimated 1 RM. The weight of the DBs was then increased 5 to 10% and a second warm-up of 3 to 5 repetitions was performed. Thereafter, weight was increased 5 to 10% incrementally until the participant could no longer complete the allotted repetitions or maintain prescribed cadence. Participants were given 5 attempts to obtain the heaviest weight they could move through a full range of motion for 5 repetitions with 2 min of rest between attempts and exercises. The same maximal strength testing procedures for both Visit 1 (bilateral) and Visit 2 (unilateral) were followed. During Visit 2, both the left and right arms were tested. Cadence was set at a 2-1-2 duty cycle for all repetitions performed [21]. A trained spotter was present at all maximal strength testing visits to ensure the safety of the participants.

### 2.4. Visits 2 and 3

#### Data Collection

Continuous ECG recordings were obtained via a 3-lead electrocardiogram (ECG). Measures of BP (i.e., SBP) were simultaneously obtained via a non-invasive BP system (Human Non-Invasive BP (NIBP) Nano; AD Instruments, Colorado Springs, CO, USA), which is considered valid and reliable [22]. HR and BP signals were collected via PowerLab data acquisition and LabChart software (AD instruments, Colorado Springs, CO, USA) and sampled at 1000 and 200 Hertz (Hz), respectively. Any ectopic beats, noise, and artifacts on the ECG were manually replaced with interpolated adjacent R wave values [23]. Following, HR signals were imported into WinCPRS (Absolute Aliens, Turku, Finland) for generation and analysis of R-R intervals in the frequency (TP, HF power, LF power, LF:HF ratio) and time (RMSSD) domains. Frequency domain measures were generated with a Fast Fourier transformation [10].

BRS was assessed using the Sequence Method according to Samora, et al. [24]. It is suggested that BRS derived from this method is representative of vagal activity (i.e., neural gain) [25]. BRS was determined from 3 consecutive heart beats in which progressive increases or decreases in SBP were followed by a lengthening or shortening in the RR interval. The threshold change was set at 5 ms for HR intervals and 1 mmHg for SBP. Only sequences with correlations ≥ 0.80 were accepted [12]. BRS was determined for all combined sequences (Gain_total_), and also separately upward (Gain_up_) and downward (Gain_down_).

Participants completed an UB ARE protocol adapted from that of Fahs, et al. [21]. In the present study, following collection of resting measures and a brief warm-up, participants completed 4 sets of 5 repetitions at their 5 RM on the DB bench press followed by 4 sets of 10 repetitions at the 10 RM on the biceps curl. Participants were given 2 min of rest between sets and exercises. During the unilateral UB ARE, participants performed the repetitions in either the right or left limb first, which was randomized, then were given approximately 2 min of rest prior to performing the next set of repetitions in the same limb. This procedure was followed to ensure that time-under-tension matched that of the bilateral UB ARE condition. The same cadence (2-1-2) as previous was used for all repetitions during data collection visits. If participants could not complete the allotted repetitions, a drop set was initiated whereby weight was reduced 2.2 kg per limb until the participant could complete the allotted repetitions with proper form and cadence. A spotter was present to ensure safety, proper form, and cadence. All repetitions, including load performed, were recorded for determination of total workload (sets × weight × repetitions). Following the UB ARE participants returned to the supine position and were assessed at 10- and 30-min following the ARE.

### 2.5. Statistical Analyses

Independent -samples tests were conducted to determine differences in total workload between UB ARE conditions (bilateral, unilateral), as well as differences in characteristics between men and women. Prior to tests of Analysis of Variance (ANOVA), all data were examined for outliers and assumptions of ANOVA using IBM SPSS Version 21 (Armonk, NY, USA). Tests of Normality were conducted via Shapiro–Wilk for all variables. Violations of sphericity were adjusted utilizing a Greenhouse–Geisser correction. Variables that were not normally distributed (TP, HF power, LF power, LF:HF ratio, and RMSSD) underwent logarithmic (ln) transformation. A 2 × 3 Repeated Measures ANOVA was used to examine differences between conditions (bilateral, unilateral) on the repeated factor of time (Rest, 10- and 30-min) on cardiac ANS function as measured by HRV (HR, ln TP, ln HF power, ln LF power, ln LF:HF ratio, and ln RMSSD), BP (SBP), and BRS (Gain_total_, Gain_up_, and Gain_down_). Partial eta squared (*η_p_^2^*) was used to assess the effect size of each dependent variable. Significant interactions and main effects were further analyzed using paired t-tests with a Holm–Bonferroni correction factor to correct for alpha inflation [26]. All results are presented as mean ± standard deviation (SD). Sample size was calculated a priori using G*Power version 3.1.9.2 [27]. Given a desired power of at least 80%, an alpha of 0.05, and a range of effect sizes for the HR (Cohen’s f = 1.056) ln HF power (Cohen’s f = 2.156), and ln RMSSD (Cohen’s f = 0.949), calculated from data by Tai, et al. [3], a total sample size of 4 to 6 participants completing each condition was determined.

## 3. Results

### 3.1. Participant Characteristics and UB ARE Workloads

Participant characteristics and UB ARE workloads are presented in Table 1 and Table 2, respectively. Men (*n* = 7) were significantly (*p* ≤ 0.001) taller and heavier than Women (*n* = 7). There were no significant (*p >* 0.05) differences between bilateral and unilateral UB ARE workloads.

### 3.2. HRV Following UB ARE

Measures of cardiac ANS function relative to HRV are presented in Table 3. There were no significant (*p* > 0.05) condition by time interactions for any HRV measure. However, there were significant main effects of time for HR (F (2,26) = 7.216, *p* = 0.004, *n_p_*^2^ = 0.38), ln TP (F (2,26) = 4.58, *p* = 0.02, *n_p_*^2^ = 0.26), ln HF power (F (2,26)) = 10.37, *p* ≤ 0.001, *n_p_*^2^ = 0.44), and ln RMSSD (F (2,26) = 12.97, *p* ≤ 0.001, *n_p_*^2^ = 0.49). HR was increased at all time points during recovery and did not return to resting values within 30 min. The ln TP, ln HF power, and ln RMSSD were collectively decreased at all time points during recovery and did not return to resting levels in 30 min. There were no significant (*p* > 0.05) main effects of time for ln LF power or ln LF:HF ratio.

### 3.3. BRS Following UB ARE

Cardiac ANS function relative to BP and BRS are presented in Table 4. There were no significant (*p* > 0.05) condition by time interactions for SBP or BRS measures. However, there were significant main effects of time for BRS relative to Gain_total_ (F (2,26) = 6.17, *p* = 0.006, *n_p_*^2^ = 0.32), Gain_up_ (F (2,26) = 10.21, *p* ≤ 0.001, *n_p_*^2^ = 0.46) and Gain_down_ (F (2,26) = 8.067 (*p* = 0.002, *n_p_*^2^ = 0.40), such that Gain_up_ increased at 10 and 30 min compared to Rest. Additionally, there was an increase in Gain_total_ and Gain_down_ at 30 min compared to Rest. There were no significant (*p* > 0.05) main effects of time for SBP.

## 4. Discussion

This study investigated the effects of bilateral and unilateral UB ARE on cardiac ANS function in healthy young, moderately active individuals. The primary findings of the present study are somewhat in disagreement with the study hypotheses, and with those of Moreira, et al. [7]. The work by Moreira, et al. [7] previously suggested that bilateral and unilateral UB ARE produce different cardiovascular responses. Instead, data in the present study demonstrate that bilateral and unilateral UB ARE produce similar alterations in cardiac ANS function, such that vagal tone was attenuated for at least 30 min during recovery as demonstrated by reductions in measures of HRV and BRS.

The observed changes in cardiac ANS function following UB ARE are largely in agreement with the current literature [2,3,4]. Kingsley, et al. [2] and Tai, et al. [3] also reported an increase in HR up to 30 min during recovery from UB ARE. Further, we reported a reduction in ln TP following both bilateral and unilateral UB ARE for at least 30 min. This finding was similar to Tai, et al. [3] who also reported reductions following UB ARE consisting of 4 sets of 8 at 70% 1 RM on the barbell bench press exercise, but these attenuations were greater in magnitude (−4% vs. −14%, respectively). Both these findings are disparate from Kingsley, et al. [2] who reported no change in ln TP following chest press and seated row exercise for 3 sets of 10 at the 10 RM. Nevertheless, Tai, et al. [3] and Kingsley, et al. [2], did report reductions in other HRV measures at 30 min, namely ln HF power. However, while ln HF power in this study was attenuated (−7%), Kingsley, et al. [2] and Tai, et al. [3] reported larger reductions (−13% and −23%, respectively) at this time point further suggesting greater vagal withdrawal. Tai, et al. [3] also reported increases in the ln LF: HF ratio, which was not demonstrated in the present study nor Kingsley, et al. [2]. These collective differences may be attributed to several differences between the UB ARE protocols mentioned such as active muscle groups, use of machines or free weights, and load. Further, unlike the present study, Kingsley, et al. [2], Tai, et al. [3], and De Freitas, et al. [4] did not control for repetition cadence. Previous studies have suggested that greater time under tension may accelerate vagal withdrawal and delay its recovery [28]. Collectively, it is unclear how each of these variables alone or in combination would influence HRV measures. Further, this is the first study to utilize a compound DB exercise, such as the bench press, regarding changes in cardiac ANS function as all other studies have utilized either free weights, or plate loaded weight machines. Given our findings in comparison to those in the literature, DB exercise compared to free weight or plate loaded exercise, may result in an attenuated vagal withdrawal independent of load. However, this is speculative as load was only reported by Kingsley, et al. [2] and not by Tai, et al. [3]. Nevertheless, it is evident that both bilateral and unilateral UB ARE alter cardiac function during recovery by decreasing HRV measures representative of vagal tone.

Similar to the present study, Kingsley, et al. [2], Tai, et al. [3] and De Freitas, et al. [4] also report reductions in the RMSSD. However, while the data in the present study reported reductions in the ln RMSSD of 16% and ~6% at 10 and 30 min, respectively. Kingsley, et al. [2], Tai, et al. [3], and De Freitas, et al. [4] again report individual reductions in the ln RMSSD or RMSSD that are larger in magnitude. Mainly, Kingsley, et al. [2] reported reductions of −11% at 30 min, and Tai, et al. [3], and De Freitas, et al. [4] reported reductions of −23% and −33% at 10 min, −24% and −18% at 30 min, respectively. Again, these dissimilarities may be attributed to differences in the UB RE protocol. The protocol by De Freitas, et al. [4] was lower in intensity (i.e., 3 sets at 65% 1 RM) with similar rest length (i.e., 90 s), but participants performed 3 upper body exercises: chest press, row, and biceps curl using a combination of free weights and weight machines. Additionally, caution should be made in direct comparison of the RMSSD reported by De Freitas, et al. [4] to the present study as they did not perform a logarithmic transformation. In summary, these findings suggest that vagal recovery may be mediated by parameters of the UB ARE itself (e.g., rest period length, the number of exercises performed, and cadence), but warrants further investigation.

In this study, changes in SBP were unremarkable. This is similar to the findings of Tai, et al. [3]. However, there were significant reductions in BRS in the present study, but these changes were similar between bilateral and unilateral UB ARE. The findings regarding BRS recovery are novel as no other study to date has reported these measures following UB ARE alone. Nevertheless, a few studies have examined changes in BRS following full-body ARE and report that BRS was also reduced at similar time points [29,30]. For example, Kingsley, et al. [29] reported reductions in BRS following full-body ARE consisting of free weight exercise (e.g., squat, bench press, deadlift) performed for 3 sets of 10 at 70% 1 RM. Additionally, Heffernan, et al. [30] reported reductions in BRS at similar time points following a combination of full-body free-weight and machine weight exercise (e.g., bench press, bent-over row, leg extension, leg curl, military press, biceps curl, close-grip bench press, and abdominal crunch) for 3 sets of 10 at the 10 RM. Unfortunately, direct comparisons in BRS changes across study protocols cannot be made from the present study due to differences in reporting of BRS. However, the present study suggests that compared to recovery of HRV, the recovery of BRS is delayed. That is, while HRV measures at 30 min began to return towards baseline the same was not true for measures of BRS. Therefore, while UB ARE reduces BRS, the magnitude of this difference compared to full--body ARE is unknown and its relation to HRV recovery is unclear.

### Limitations

There are a few limitations in the present study. Specifically, this study included both Men and Women, whom had differences in terms of height and weight, with Men being both taller and heavier. However, these characteristics were not run as a covariate due to the failed assumption of linearity between the covariate and measures of cardiac ANS function. Additionally, sex differences could not be determined in this study due to insufficient power. Previous literature has reported no sex differences for HRV or BRS in resistance-trained individuals [29], but whether this is also true in moderately active individuals is unclear. In addition, the unilateral UB ARE involved greater core stabilization compared to the bilateral UB ARE. Cardiovascular responses are muscle mass dependent [8,9], thus increased abdominal muscle involvement may have contributed to the findings of the present study. Collectively, these factors during the unilateral UB ARE may have allowed for similar responses to that of the bilateral UB ARE. Further, the Valsalva maneuver was not controlled for during the UB ARE, which may have independently influenced measures of cardiac ANS function. Lastly, the results of this study are limited to young, healthy resistance-trained individuals.

## 5. Conclusions

In conclusion, the results of this study do not support the initial hypotheses as there were no differences in HRV or BRS measures at any time point between bilateral and unilateral UB ARE. Nevertheless, these measures indicate that cardiac ANS function is altered for at least 30 min. Given that an acute bout of resistance exercise reduces cardiac ANS function for this period of time, future studies may wish to investigate time to recovery following either of these modalities, as well as with additional exercises.

## Figures and Tables

**Table 1:** Participant characteristics (*n* = 14; Men = 7, Women = 7).

	Men (*n* = 7)	Women (*n* = 7)
**Age (year)**	23 ± 3	23 ± 3
**Height (m)**	1.8 ± 0.1 ^§^	1.7 ± 0.0
**Weight (kg)**	81.9 ± 10.1 ^§^	67.8 ± 10.7
**BMI (kg/m^2^)**	25.9 ± 3.0	24.5 ± 3.0

Data presented are mean ± SD. BMI = body mass index. ^§^ Significantly different from Women (*p* ≤ 0.001).

**Table 2:** Bilateral and unilateral upper body acute resistance exercise workloads (*n* = 14; Men = 7; Women = 7).

	Workload (kg)
**Bilateral DB Bench Press**	2189 ± 954
**R, Unilateral DB Bench Press**	2105 ± 1081
**L, Unilateral DB Bench Press**	2218 ± 1039
**Bilateral DB Biceps Curl**	1996 ± 820
**R, Unilateral DB Biceps Curl**	1998 ± 821
**L, Unilateral DB Biceps Curl**	1886 ± 744

Data presented are mean ± SD. DB = Dumbbell; L = Left Arm; R = Right Arm.

**Table 3:** Measures of heart rate variability at rest and during recovery from bilateral and unilateral upper body acute resistance exercise (*n* = 14; Men: 7; Women: 7).

	Bilateral (*n* = 14)			Unilateral (*n* = 14)		
	Rest	10 min	30 min	Rest	10 min	30 min
**HR (bpm)**	54 ± 5	68 ± 10 *	61 ± 8 *^,†^	56 ± 7	68 ± 12 *	62 ± 9 *^,†^
**TP (ln ms^2^)**	8.4 ± 0.8	7.4 ± 0.9 *	8.0 ± 0.8 *^,†^	8.4 ± 0.8	7.6 ± 1.0 *	8.1 ± 0.9 *^,†^
**HF (ln ms^2^)**	7.5 ± 1.0	6.2 ± 1.1 *	7.1 ± 0.9 *^,†^	7.5 ± 1.0	6.4 ± 1.4 *	6.9 ± 1.2 *^,†^
**LF (ln ms^2^)**	6.7 ± 0.8	5.6 ± 1.0	6.4 ± 1.0	6.6 ± 0.9	5.9 ± 0.9	6.4 ± 0.9
**LF/HF ratio (ln)**	3.8 ± 0.8	4.0 ± 0.8	3.9 ± 0.9	3.7 ± 0.8	4.1 ± 0.9	4.1 ± 0.7
**RMSSD (ln ms^2^)**	4.3 ± 0.6	3.5 ± 0.6 *	4.1 ± 0.5 *^,†^	4.2 ± 0.5	3.6 ± 0.7 *	4.0 ± 0.7 *^,†^

Data presented are mean ± SD. HF = High-Frequency power; HR = Heart Rate; LF = Low-Frequency power; RMSSD = Root Mean Square of Successive Differences Between Normal-to-Normal Heart Beats; TP = Total Power. * Significantly different from Rest (*p* ≤ 0.05), ^†^ Significantly different from 10 min *(p* ≤ 0.05).

**Table 4:** Measures of blood pressure and baroreflex sensitivity at rest and during recovery from bilateral and unilateral upper body acute resistance exercise (*n* = 14; Men: 7; Women: 7).

	Bilateral (*n* = 14)			Unilateral (*n* = 14)		
	Rest	10 min	30 min	Rest	10 min	30 min
**SBP (mmHg)**	114 ± 7	114 ± 9	113 ± 7	114 ± 7	114 ± 8	115 ± 8
**Gain_total_ (ms∙mmHg)**	16 ± 10	21 ± 15	28 ± 13 *	19 ± 11	25 ± 13	31 ± 20 *
**Gain_up_ (ms∙mmHg)**	6 ± 5	12 ± 8 *	12 ± 6 *	10 ± 7	14 ± 8 *	16 ± 11 *
**Gain_down_ (ms∙mmHg)**	8 ± 5	11 ± 7	14 ± 8 *	10 ± 7	12 ± 7	16 ± 10 *

Data presented are mean ± SD SBP = Systolic Blood Pressure. * Significantly different from Rest (*p* ≤ 0.05).

## Data Availability

Data presented in this study are available upon request from the corresponding author. The data are not publicly available due to privacy/ethical concerns.
